# Complemental Value of Microstructural and Macrostructural MRI in the Discrimination of Neurodegenerative Parkinson Syndromes

**DOI:** 10.1007/s00062-023-01377-w

**Published:** 2024-01-30

**Authors:** Nils Schröter, Philipp G. Arnold, Jonas A Hosp, Marco Reisert, Michel Rijntjes, Elias Kellner, Wolfgang H. Jost, Cornelius Weiller, Horst Urbach, Alexander Rau

**Affiliations:** 1 Department of Neurology and Clinical Neuroscience, Medical Center—University of Freiburg, Faculty of Medicine, University of Freiburg, Freiburg, Germany; 2 Department of Neuroradiology, Medical Center—University of Freiburg, Faculty of Medicine, University of Freiburg, Breisacher Str. 64, 79106 Freiburg, Germany; 3 Department of Diagnostic and Interventional Radiology, Medical Center—University of Freiburg, Faculty of Medicine, University of Freiburg, Freiburg, Germany; 4 Department of Medical Physics, Medical Center—University of Freiburg, Faculty of Medicine, University of Freiburg, Freiburg, Germany; 5 Department of Stereotactic and Functional Neurosurgery, Medical Center—University of Freiburg, Faculty of Medicine, University of Freiburg, Freiburg, Germany; 6 Parkinson-Klinik Ortenau, Wolfach, Germany

**Keywords:** Parkinson’s disease, Progressive supranuclear palsy, Multiple system atrophy, Diffusion magnetic resonance imaging, Diffusion tensor imaging

## Abstract

**Purpose:**

Various MRI-based techniques were tested for the differentiation of neurodegenerative Parkinson syndromes (NPS); the value of these techniques in direct comparison and combination is uncertain. We thus compared the diagnostic performance of macrostructural, single compartmental, and multicompartmental MRI in the differentiation of NPS.

**Methods:**

We retrospectively included patients with NPS, including 136 Parkinson’s disease (PD), 41 multiple system atrophy (MSA) and 32 progressive supranuclear palsy (PSP) and 27 healthy controls (HC). Macrostructural tissue probability values (TPV) were obtained by CAT12. The microstructure was assessed using a mesoscopic approach by diffusion tensor imaging (DTI), neurite orientation dispersion and density imaging (NODDI), and diffusion microstructure imaging (DMI). After an atlas-based read-out, a linear support vector machine (SVM) was trained on a training set (*n* = 196) and validated in an independent test cohort (*n* = 40). The diagnostic performance of the SVM was compared for different inputs individually and in combination.

**Results:**

Regarding the inputs separately, we observed the best diagnostic performance for DMI. Overall, the combination of DMI and TPV performed best and correctly classified 88% of the patients. The corresponding area under the receiver operating characteristic curve was 0.87 for HC, 0.97 for PD, 1.0 for MSA, and 0.99 for PSP.

**Conclusion:**

We were able to demonstrate that (1) MRI parameters that approximate the microstructure provided substantial added value over conventional macrostructural imaging, (2) multicompartmental biophysically motivated models performed better than the single compartmental DTI and (3) combining macrostructural and microstructural information classified NPS and HC with satisfactory performance, thus suggesting a complementary value of both approaches.

**Supplementary Information:**

The online version of this article (10.1007/s00062-023-01377-w) contains supplementary material, which is available to authorized users.

## Introduction

Parkinson’s disease (PD) is the fastest growing neurological disorder and a leading source of disability [1]. Atypical Parkinson syndromes comprise multiple system atrophy (MSA), progressive supranuclear palsy (PSP) and corticobasal degeneration. As clinical symptoms of these diseases can be difficult to distinguish especially in the early stages, correct diagnosis is highly dependent on the experience of the clinician [2]. Thus, up to 20–25% of patients with PD and approximately 30% of patients with MSA and PSP are initially misdiagnosed based only on clinical characteristics [3].

While 18‑F fluorodeoxyglucose positron emission tomography–computed tomography (FDG PET-CT) has high diagnostic accuracy in the differential diagnosis of neurodegenerative Parkinson syndromes (NPS), it is currently only available at centers specialized in neuroimaging and neurodegenerative diseases [3]. I^123^-ioflupane single-photon emission computed tomography (SPECT) primarily delineates neurodegenerative and non-neurodegenerative Parkinson syndromes but does not distinguish between different Parkinson syndromes [4]. Currently, a widely accessible serum marker is not available [5]. Because of its broad use, noninvasiveness, and comparatively low cost, MRI represents a promising technique to fill the diagnostic gap.

In suspected NPS, MRI is typically employed to exclude structural abnormalities such as malignancies, small vessel disease, strategic infarcts, or hydrocephalus [6]. Nevertheless, MRI can contribute to delineating Parkinson syndromes as the various NPS each exhibit pathognomonic patterns of neurodegeneration [7]. As microstructural precede macrostructural cerebral changes and thus offer a potentially earlier and possibly more accurate diagnosis [8–10], several different methods have been evaluated in the differential diagnosis of NPS in recent years. Mesoscopic approaches that approximate the brain’s microstructure in vivo were in vivo were employed to improve the diagnostic value of MRI. The diffusion tensor imaging (DTI) parameters were applied to identify NPS-related changes [11–14]. For example, an elevated nigral mean diffusivity and reduced fractional anisotropy were observed compared to healthy subjects; however, the interpretability of DTI metrics is constrained as they are limited to information on the orientation and isotropy of diffusivity [15]. Compared to DTI, biophysically motivated models, such as neurite orientation dispersion and density imaging (NODDI) and diffusion microstructure imaging (DMI) provide a more specific and interpretable approximation of the microstructure [16–18], e.g., observing an increased nigral-free water fraction in neurodegenerative Parkinson syndromes [19, 20]. In contrast to NODDI, DMI is not restricted to hard a priori assumptions, therefore is more suitable to assess pathologically altered microstructures [17] and has already been successfully applied in clinical research [14, 20–23].

In this retrospective study, we analyzed and compared the diagnostic value of macrostructural and microstructural MRI metrics to differentiate patients with PD, MSA, and PSP and healthy controls (HC) and developed a fully automatic algorithm for assessing NPS. We hypothesized that (1) MRI parameters that approximate the cerebral microstructure using a mesoscopic approach perform significantly better in delineating NPS and HC than those derived from macrostructural imaging, (2) MRI parameters based on multicompartmental techniques perform better than single compartmental DTI and (3) the combination of these approaches performs best.

## Material and Methods

### Participants

**Fig. 1:**
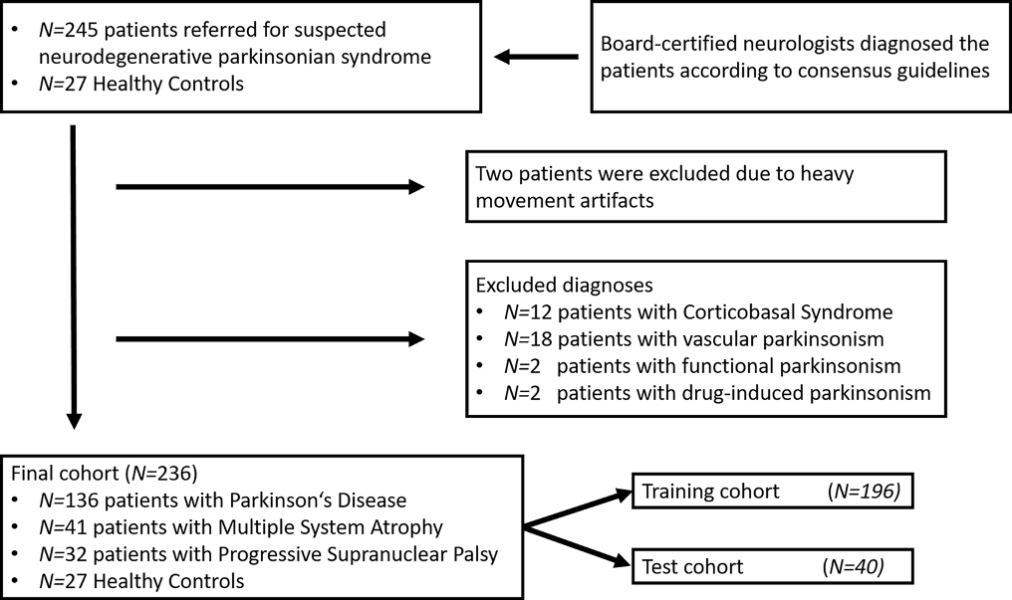
Flowchart for patient inclusion and exclusion

This retrospective single center cross-sectional study included consecutive patients with NPS according to current consensus diagnostic criteria [24–26] who underwent MRI for the differential diagnosis of NPS between January 2018 and December 2021. Disease was staged according to Hoehn and Yahr [27]. Patients with suspected corticobasal degeneration and dementia with Lewy bodies were excluded from this study due to the small number of cases and etiological heterogeneity. We included age-matched and sex-matched HC. The inclusion workflow is shown in Fig. 1.

For training and subsequent validation of our SVM algorithm, we randomly split our cohort into a training and test subset (4:1) matched in terms of age, sex, and diagnoses.

The study was approved by the Institutional Review Board (EK22/20) and carried out in accordance with the Declaration of Helsinki and its later amendments. The need for written informed consent was waived.

### MRI Acquisition

MRI was performed with a 3-Tesla scanner (MAGNETOM Prisma, Siemens Healthcare, Erlangen, Germany) with a 64-channel head and neck coil and included high resolution T1w and multishell diffusion MRI data. T1-weighted (T1w) images were acquired with three-dimensional (3D) magnetization-prepared 180° radiofrequency pulses and a rapid gradient-echo (MP-RAGE) sequence (repetition time: 2500 ms; echo time: 2.82 ms; flip angle: 7°, TI = 1100 ms; GRAPPA factor = 2; 1.0 mm isotropic voxels; 192 contiguous sagittal slices). The diffusion-weighted sequence was acquired with the following parameters: axial orientation, 42 slices, voxel size 1.5 × 1.5 × 3 mm^3^, TR 2800 ms, TE 88 ms, band width 1778 Hz, flip angle 90°, simultaneous multiband acceleration factor 2, GRAPPA factor 2, 58 diffusion-encoding gradient directions per shell with b‑factors 1000 and 2000 s/mm^2^, and 15 non-diffusion weighted images (interleaved during diffusion-encoding directions); this resulted in a total of 131 images.

### Spatial Normalization and Calculation of Tissue Probability Values

All data processing was carried out on a local instance of the NORA platform (www.nora-imaging.org). To obtain a macrostructural measure of atrophy, T1w-imaging datasets were automatically segmented into white and gray matter using CAT12 (http://www.neuro.uni-jena.de/cat/). Diffusion-weighted images were rigidly coregistered to the T1w images using the SPM toolbox.

### Calculation of Diffusion MRI Parameters

Preprocessing of diffusion-weighted images included a denoising step [28], followed by the correction of Gibbs ringing artifacts [29] and final upsampling to an isotropic resolution of 1.5 mm^3^.

The DTI measures, i.e., fractional anisotropy (FA), mean diffusivity (mD), radial diffusivity (rD), and axial diffusivity (aD), were obtained from b = 0 and 1000 s/mm^2^ images using a publicly available open-source toolbox (https://www.uniklinik-freiburg.de/mr-en/research-groups/diffperf/fibertools.html) using the ordinary log-linear fitting.

Applied on multishell diffusion data, NODDI and DMI enable the differentiation of microstructural compartments based on a standard white matter model consisting of 3 components [16–18]: (1) the free water/CSF fraction (Viso, V‑CSF) with random unhindered molecule movement. (2) The intra-axonal volume fraction (Vic, V‑intra) with a nearly one-dimensional diffusion due to tight membrane boundaries. (3) The extra-axonal volume fraction (V-extra), characterized by restricted diffusion and corresponding to the extra-axonal cellular compartment and the extracellular matrix.

The NODDI parameters (i.e., Vic, Viso, and orientation dispersion, OD) were obtained by accelerated microstructure imaging via convex optimization (AMICO)-NODDI [30].

DMI metrics based on a three-compartment diffusion model (i.e., V‑CSF, V‑intra, and V-extra) were estimated using a Bayesian approach and machine learning techniques [17]. In fact, NODDI and DMI are based on a very similar mode, whereas the NODDI approach, relies on fixes for certain parameters of the model (intra-axonal and extra-axonal diffusion coefficients), while DMI relaxes these hard constraints by using broad prior distributions.

### Extraction of Macrostructural and Microstructural Imaging Features

**Fig. 2:**
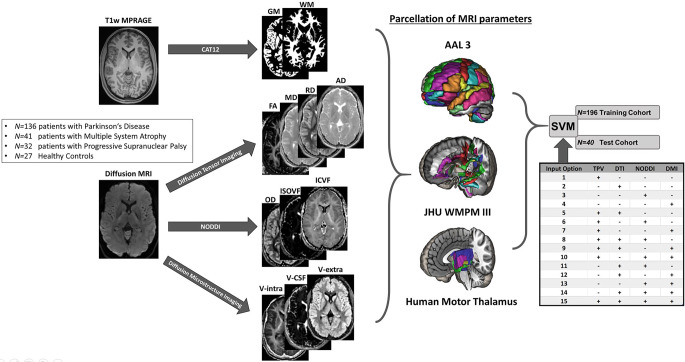
Schematic of data processing and input into the support vector machine (SVM). *GM* gray matter tissue probability value, *WM* white matter tissue probability value, *NODDI* neurite orientation dispersion and density imaging, *DTI *diffusion tensor imaging, *DMI *diffusion microstructure imaging, *TPV *tissue probability value

The parameter maps of DTI, NODDI, and DMI were separated into gray and white matter using a CAT12-derived tissue probability value (TPV) threshold of 0.4. For this, the TPV provides the probability of a voxel to be attributed to gray or white matter. From this, only the gray matter compartment was read for the AAL3 atlas and only the white matter part for the JHU WMPM III atlas (Fig. 2; [31, 32]). In addition, we extracted the MRI metrics from the Human Motor Thalamus atlas [33].

### SVM Training

A linear support vector machine (SVM) was trained and optimized in respect of the area under the receiver operating characteristic curve (AUC-ROC) in a one-vs.-rest (OVR) classifier for PD, MSA, PSP, and HC. The diagnostic performance of the SVM was compared with respect to different inputs, i.e., TPV, DTI, NODDI, and DMI individually and in combination.

The SVM was developed in the Python (version 3.8.5) package “Scikit-learn” (version 0.23.2). The atlas-derived microstructural and macrostructural parameters served as input for the linear SVM. We investigated the diagnostic value of 638 TPV-derived features, 2124 features obtained by DTI, 1584 by NODDI, and 1593 by DMI resulting in a total of 5939 features with data available in all participants. To address the considerably different group sizes, class_weight was set to ’balanced’. The class_weight argument can be specified as a model hyperparameter and via a dictionary defines each class label and the weighting to apply to the C value in the calculation of the soft margin. Different combinations of input parameters for the training subset were tested. Using the Scikit-learn StandardScaler, the input parameters were normalized to mean 0 and standard variance.

Prior to training, the input parameters were sorted with respect to maximum marginal diversity [34]. We chose this approach because of the relatively small subgroup sizes in relation to the maximum number of input features. For this, normalized values of a given input feature are distributed into 20 equally spaced bins separately for each group. Subsequently, the Kullback-Leibler divergence for the resulting group-wise histogram is calculated as a measure of the difference between the distributions. To obtain the best combination of the linear SVM parameter C and the number of input parameters, different linear SVMs were trained in a grid search approach with C varying between 0.01 and 100 and the number of input parameters varying between the top 5–40%, fivefold cross-validated and compared with respect to the area under the curve (AUC). The maximum number of input parameters was set to be below 40% to reduce noise and prevent overfitting.

### Statistical Analysis

Statistical analysis was performed using R (version 4.1.0, https://www.R-project.org/). Data are presented as the mean and standard deviation for continuous variables and as absolute frequencies and percentages for categorical variables. We compared demographic and clinical characteristics of patient and control groups with analysis of variance (ANOVA) followed by Tukey’s honest significance test, or the Kruskal-Wallis test with post hoc pairwise, Bonferroni-Holm corrected, Wilcoxon test. The AUC-ROC and DeLong’s test were employed to investigate and compare the discriminative power of the different SVM inputs. The significance threshold was set to *p* < 0.05.

## Results

### Participants

We report on MRI data from (I) 209 NPS patients (PD, 136; MSA, 41; PSP, 32) who underwent routine MRI between January 2018 and December 2021, and (II) 27 age-matched and sex-matched HC of an inhouse control cohort. Results of [18F]-FDG-PET were available in 179/209 patients, supportive of the clinical diagnosis. The inclusion workflow is given in Fig. 1 and further characteristics are provided in Table 1.

**Table 1:** Demographic and clinical characteristics of patient groups

	Healthy controls (*n* = 27)	Parkinson’s disease (*n* = 135)	Multiple system atrophy (*n* = 40)	Progressive supranuclear palsy (*n* = 32)
*Sex*				
Female (%)	56	36^g^	55^g^	50
*Age (years)*				
Mean (SD)	65 (8)^a^	66 (9)^b^	65 (10)^c^	73 (7)^a, b, c^
*Hoehn and Yahr stage* [27]				
Median [min–max]	–	3.0 [1.0–5.0]^d^	3.0 [2.0–5.0]^d^	3.0 [2.0–4.0]
*UPDRS III in OFF-State*				
Mean (SD)	–	46.1 (17.9)	49.7 (18.3)	41.1 (16.2)
*Disease duration (years)*				
Median [min–max]	–	9.0 [0.5–26.1]^e,f^	3.6 [0.4–19.7]^e,h^	2.61 [0.1–6.2]^f,h^

Hoehn and Yahr stage at MRI imaging was missing for eight patients with MSA and one patient with PSP. UPDRS III in OFF-State was missing in 11 patients with PD, 6 patients with MSA and 11 patients with PSP

*HC* healthy controls, *PD* Parkinson’s disease, *MSA* multiple system atrophy, *PSP* progressive supranuclear palsy, *SD* standard deviation, *UPDRS III in OFF-State* unified Parkinson’s disease rating scale motor part 3 (off medication)

^a–e^Significance of pairwise comparisons: a, b, c, e, f *p* < 0.001; d, h *p* < 0.01; g *p* < 0.05

The training cohort (*n* = 196: 115 PD, 35 MSA, 25 PSP, 21 HC) matched the test subset (*n* = 40: 21 PD, 6 MSA, 7 PSP, 6 HC) in terms of age, sex, and diagnoses (each *p* > 0.05).

### Comparison of Different SVM

In general, TPV alone performed inferior to each dMRI-derived metric alone in both the training and test cohorts. Using the DeLong’s test for the training cohort, we found a significant superiority of the dMRI-derived metrics alone compared with TPV in the delineation of patients with PD (TPV vs. DTI, *p* = 0.027; TPV vs. NODDI, *p* = 0.004; TPV vs. DMI, *p* = 0.047), while none of the dMRI inputs performed superior to another as given in Supplementary Table 1. This finding, however, did not reach significance in the test cohort (Supplementary Table 2).

**Fig. 3:**
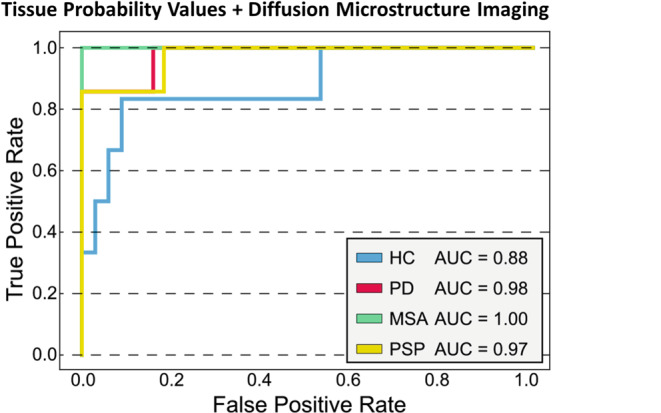
Receiver operating characteristic curves for the combination of tissue probability values and diffusion microstructure imaging as the best performing combination to classify neurodegenerative Parkinson syndromes and healthy controls in the test cohort. *HC* healthy controls, *PD* Parkinson’s disease, *MSA* multiple system atrophy, *PSP* progressive supranuclear palsy, *AUC* area under the curve

**Fig. 4:**
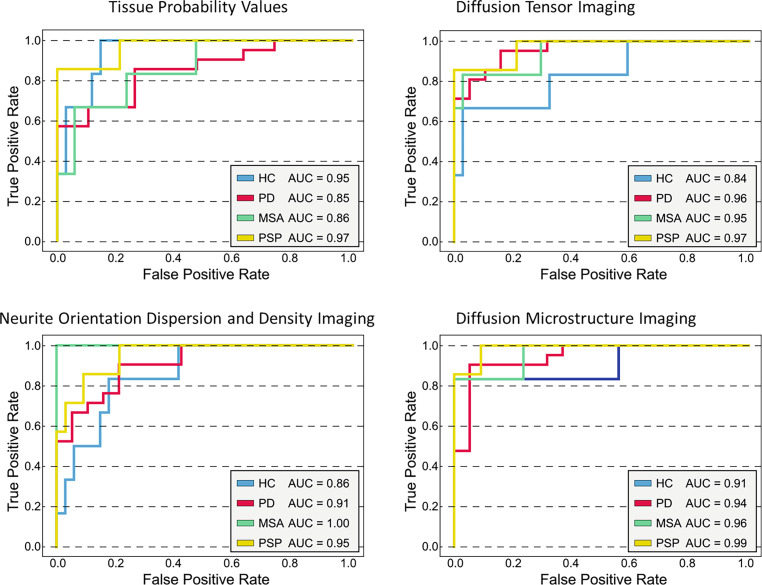
Receiver operating characteristic curves for each investigated input separately and diagnostic performance in the test cohort as given by the area under the curve (AUC) to classify neurodegenerative Parkinson syndromes and healthy controls. Top left depicts the performance for the tissue probability values as input, top right for diffusion tensor imaging, bottom left for neurite orientation dispersion and density imaging, and bottom left for diffusion microstructure imaging. *HC* healthy controls, *PD* Parkinson’s disease, *MSA* multiple system atrophy, *PSP* progressive supranuclear palsy

**Fig. 5:**
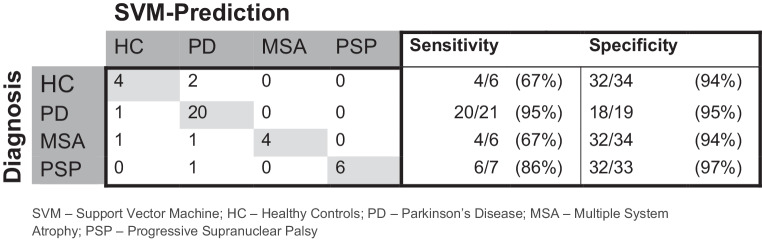
Confusion matrix for the combination of the tissue probability values and diffusion microstructure imaging metrics as the best performing input in the test cohort. *SVM* support vector machine, *HC* healthy controls, *PD* Parkinson’s disease, *MSA* multiple system atrophy, *PSP* progressive supranuclear palsy

The overall best diagnostic performance in the training cohort was revealed for the combination of TPV, DTI, and NODDI (see Supplementary Table 3). The corresponding OVR-ROC-AUC was 0.95 for HC, 0.94 for PD, 0.99 for MSA, and 0.96 for PSP. In the test cohort, the combination of DMI and TPV performed best and correctly classified 85% of the participants (i.e. the patients and healthy controls) and 88% of the patients (Fig. 3). The corresponding OVR-ROC-AUC was 0.88 for HC, 0.98 for PD, 1.0 for MSA, and 0.97 for PSP with sensitivities of 67% for HC, 95% for PD, 67% for MSA, and 86% for PSP. Respective specificities were 94% (HC), 95% (PD), 94% (MSA), and 97% (PSP). Upon inspection of performance of the dMRI metrics alone, we found better diagnostic performance for DMI compared with DTI or NODDI, however not reaching significance in the De Long test (AUCs of DMI 0.91 for HC; 0.94 for PD; 0.96 for MSA; 0.99 for PSP vs. DTI: 0.84 for HC; 0.96 for PD; 0.95 for MSA; 0.97 for PSP vs. NODDI: 0.86 for HC; 0.91 for PD; 1.00 for MSA; 0.95 for PSP). See Fig. 4 and Tables 2 and Fig. 5 for more details.

**Table 2:** Areas under the curve of the receiver operating characteristics in the test cohort

	Healthy controls	Parkinson’s disease	Multiple system atrophy	Progressive supranuclear palsy
TPV	0.95	0.85	0.86	0.97
DTI	0.84	0.96	0.95	0.97
TPV + DTI	0.89	0.90	1.00	0.98
NODDI	0.86	0.91	1.00	0.95
NODDI + TPV	0.86	0.96	1.00	0.97
NODDI + DTI	0.84	0.94	1.00	0.97
NODDI + DTI + TPV	0.86	0.95	1.00	0.99
DMI	0.91	0.94	0.96	0.99
DMI + TPV	0.89	0.98	1.00	0.98
DMI + DTI	0.89	0.91	1.00	0.98
DMI + DTI + TPV	0.88	0.91	1.00	0.98
DMI + NODDI	0.88	0.97	0.99	0.97
DMI + NODDI + TPV	0.87	0.97	1.00	0.97
DMI + NODDI + DTI	0.87	0.92	1.00	0.98
DMI + NODDI + DTI + TPV	0.86	0.95	1.00	0.98

*DMI* diffusion microstructure imaging, *DTI* diffusion tensor imaging, *NODDI* neurite orientation dispersion and density imaging, *TPV* tissue probability value

### Most Discriminative Regions

For the combination of DMI and TPV, the maximum marginal diversity algorithm revealed a high diversity of the group-specific marginal distribution especially for the putamen, the cerebellar peduncles, the pons, the pontine crossing tracts, the pallidum, various cerebellar regions, and the frontal white matter as given in Supplementary Table 4. In contrast, the SVM attributed high coefficient weight to widespread gray and white matter regions exceeding the areas with high maximum marginal diversity weight. In detail, cortical regions including the cingulum, temporal, and frontal cortex had a high discriminative value (Supplementary Table 4). Information on the best-performing combination (i.e., TPV, DTI, and NODDI) is provided in Supplementary Table 5.

## Discussion

In the differential diagnosis of NPS, the microstructural parameters obtained in a mesoscopic approach using diffusion MRI data outperformed the macrostructural data derived from conventional structural T1w imaging. Of note, the combination of microstructural DMI and macrostructural TPV performed best.

In a head-to-head comparison, we revealed higher accuracy of the mesoscopic approaches than T1w-derived TPV. Furthermore, multicompartmental approaches of NODDI and DMI performed better than single compartmental DTI. Although, despite better AUC in ROC analysis, this effect did not reach significance. We attribute this primarily to the fact that the differences in the already high diagnostic values are small and thus would require a much larger sample size for statistical significance. The added value of dMRI metrics is indicated, as for the input combination of DMI and TPV, we observed high variance in regional TPV metrics in the MMD, whereas the SVM relied more on DMI metrics. The value of advanced multicompartment diffusion MRI approaches compared to DTI has been demonstrated for NPS in previous studies. For example, Ogawa et al. used NODDI to more specifically interpret the alterations observed in DTI in patients with MSA [11]. A study using DMI was able to demonstrate the added diagnostic value over DTI to identify patients with PSP versus a cohort of different NPS and HC [14]. This finding is in line with the results of Mitchell et al. who observed NODDI parameter alterations in various brain regions in PD, MSA (Parkinson subtype only) and PSP compared to HC [35]. Using NODDI or DMI, evidence of neurodegeneration in PD was found in the substantia nigra [20, 36] as well as in the nigrostriatal pathway [37].

Despite the hypothesis that DMI is more suited than NODDI to capture pathological conditions in the calculation of metrics [17, 22], we did not detect significant superiority of DMI over NODDI. While the highest diagnostic value of the SVM was found for the input combination DMI + TPV, we could not prove a significant superiority of DMI over NODDI in DeLong’s test. Again, we attribute this primarily to the fact that the differences in the already high diagnostic values are small and also would require a much larger sample size for statistical significance. The same reasoning holds for the fact that the test and training cohorts differ with respect to the best-performing combination of input parameters.

The validity of the different macrostructural and microstructural inputs is supported by the fact that the maximum marginal diversity algorithm identified regions mainly typical for MSA and PSP [7] (i.e., middle and superior cerebellar peduncle, midbrain, and pons) while the SVM also assessed numerous supratentorial regions with high coefficients is explained by the fact that in PD atrophy is more subtle and less localized, thus expressing lower variance in the overall cohort while nevertheless having diagnostic value for the SVM. In line with this, the observed regions have been previously identified as pathologically altered in PD [7, 38].

When assessing the performance of the SVM, a particular challenge for all approaches was the differentiation between HC and PD as in conventional radiological reporting [39]. In the test cohort, one third of the HC subjects were erroneously assigned to the PD group. We especially attribute this to different group sizes with a large PD group leading to a potential bias with favor for PD; however, as the overall distribution of entities in our cohort reflects the expected distribution of diagnoses in clinical practice, the SVM’s performance is satisfactory. Future research should focus on evaluating this finding by including more HC.

Techniques based on artificial intelligence have previously been investigated to classify NPS using diffusion MRI. In line with our results, a study of 45 patients with PD, 20 with PSP, and 38 HC showed that the combination of DTI and macrostructural parameters performed best [40]. Of particular note is a multicenter study that demonstrated high diagnostic value in a dataset of 1002 patients with NPS and HC using free-water and free-water corrected FA with an AUC in PD vs. atypical parkinsonism of 0.96 and MSA vs. PSP of 0.93, although lacking a multiclass approach [13]. Compared to these approaches, we were able to demonstrate excellent AUCs in the test cohort; however, our accuracy in the test cohort was 83%, which is particularly driven by the HCs incorrectly assessed as PD. Potential approaches to overcome the diagnostic challenge of delineating HCs and PD might include the additional use of neuromelanin-sensitive imaging parameters [41].

A potential limitation is the accuracy of the clinical gold standard diagnosis [2], and subsequently the lack of pathological diagnoses. To mitigate this issue, diagnoses were made by two neurologists with long-standing experience in the diagnosis of movement disorders, additionally [18F]-FDG-PET was available in a large proportion of patients, strengthening the clinical diagnoses [3]. In order to determine the most suitable parameters for differentiating neurodegenerative Parkinson syndromes, it is essential to investigate larger, more statistically powered studies. This will contribute to further refining these methodologies. Validation of our single-center data must be subject to further studies in an external cohort. Other strengths are that we did not constrain our analyses to single atlas regions as input to prevent a priori assumptions and that the algorithm is capable of multiclass classification of NPS so we can furthermore validly identify HC in this process.

In conclusion, we were able to demonstrate that (1) MRI parameters that approximate microstructure using a mesoscopic approach provided substantial added value over conventional macrostructural imaging, (2) multicompartmental/biophysically motivated models performed better than the single compartmental DTI, although not reaching statistical significance and (3) NPS and HC were classified with satisfactory performance using an SVM, combining macrostructural and microstructural information, thus suggesting a complementary value of both approaches.

### Supplementary Information

Supplementary Table 1 Results of DeLong’s test within the training cohort
Supplementary Table 2 Results of DeLong’s test within the test cohort
Supplementary Table 3 Areas under the curve of the receiver operating characteristics in the training cohort
Supplementary Table 4 Atlas regions from the AAL 3 atlas for gray matter and the JHU WMPM III atlas for white matter areas with highest variance in the maximum marginal diversity and highest coefficients in the support vector machine with the input combination of Tissue Probability Values + Diffusion Microstructure Imaging

## Data Availability

Data are available from the authors upon reasonable request and approval of the ethics committee. The code is available in a public repository.

## Abbreviations

AUC-ROC: Area under the receiver operating characteristic curve
DMI: Diffusion microstructure imaging
DTI: Diffusion tensor imaging
FA: Fractional anisotropy
FDG PET-CT: Fluorodeoxyglucose positron emission tomography–computed tomography
HC: Healthy control
MMD: Maximum marginal diversity
MRI: Magnetic resonance imaging
MSA: Multiple system atrophy
NODDI: Neurite orientation and dispersion imaging
NPS: Neurodegenerative Parkinson syndromes
PD: Parkinson’s disease
PSP: Progressive supranuclear palsy
SVM: Support vector machine
TPV: Tissue probability value

## References

[1] Dorsey ER, Sherer T, Okun MS, Bloem BR (2018). The emerging evidence of the Parkinson pandemic. J Parkinsons Dis.

[2] Rizzo G, Copetti M, Arcuti S, Martino D, Fontana A, Logroscino G (2016). Accuracy of clinical diagnosis of Parkinson disease: a systematic review and meta-analysis. Neurology.

[3] Meyer PT, Frings L, Rücker G, Hellwig S (2017). 18F-FDG PET in Parkinsonism: differential diagnosis and evaluation of cognitive impairment. J Nucl Med.

[4] Buchert R, Buhmann C, Apostolova I, Meyer PT, Gallinat J (2019). Nuclear imaging in the diagnosis of clinically uncertain Parkinsonian syndromes. Dtsch Ärztebl Int.

[5] Tönges L, Buhmann C, Klebe S, Klucken J, Kwon EH, Müller T (2022). Blood-based biomarker in Parkinson’s disease: potential for future applications in clinical research and practice. J Neural Transm.

[6] Berardelli A, Wenning GK, Antonini A, Berg D, Bloem BR, Bonifati V (2013). EFNS/MDS-ES/ENS [corrected] recommendations for the diagnosis of Parkinson’s disease. Eur J Neurol.

[7] Saeed U, Lang AE, Masellis M (2020). Neuroimaging advances in Parkinson’s disease and atypical Parkinsonian syndromes. Front Neurol.

[8] Canu E, Agosta F, Riva N, Sala S, Prelle A, Caputo D (2011). The topography of brain microstructural damage in amyotrophic lateral sclerosis assessed using diffusion tensor MR imaging. AJNR Am. J. Neuroradiol..

[9] Bai X, Guo T, Chen J, Guan X, Zhou C, Wu J (2022). Microstructural but not macrostructural cortical degeneration occurs in Parkinson’s disease with mild cognitive impairment. NPJ Parkinsons Dis.

[10] Zhuang L, Sachdev PS, Trollor JN, Reppermund S, Kochan NA, Brodaty H (2013). Microstructural white matter changes, not hippocampal atrophy, detect early amnestic mild cognitive impairment. PLoS One.

[11] Ogawa T, Hatano T, Kamagata K, Andica C, Takeshige-Amano H, Uchida W (2021). White matter and nigral alterations in multiple system atrophy-parkinsonian type. NPJ Parkinsons Dis.

[12] Spotorno N, Hall S, Irwin DJ, Rumetshofer T, Acosta-Cabronero J, Deik AF (2019). Diffusion tensor MRI to distinguish progressive supranuclear palsy from α-synucleinopathies. Radiology.

[13] Archer DB, Bricker JT, Chu WT, Burciu RG, McCracken JL, Lai S (2019). Development and validation of the automated imaging differentiation in parkinsonism (AID-P): a multicentre machine learning study. Lancet Digital Health.

[14] Rau A, Jost WH, Demerath T, Kellner E, Reisert M, Urbach H (2022). Diffusion microstructure imaging in progressive supranuclear palsy: reduced axonal volumes in the superior cerebellar peduncles, dentato-rubro-thalamic tracts, ventromedial thalami, and frontomesial white matter. Cereb Cortex.

[15] Kamiya K, Hori M, Aoki S (2020). NODDI in clinical research. J Neurosci Methods.

[16] Zhang H, Schneider T, Wheeler-Kingshott CA, Alexander DC (2012). NODDI: practical in vivo neurite orientation dispersion and density imaging of the human brain. Neuroimage.

[17] Reisert M, Kellner E, Dhital B, Hennig J, Kiselev VG (2017). Disentangling micro from mesostructure by diffusion MRI: a Bayesian approach. Neuroimage.

[18] Novikov DS, Veraart J, Jelescu IO, Fieremans E (2018). Rotationally-invariant mapping of scalar and orientational metrics of neuronal microstructure with diffusion MRI. Neuroimage.

[19] Rau A, Hosp JA, Rijntjes M, Weiller C, Kellner E, Berberovic E (2023). Cerebellar, not nigrostriatal degeneration impairs dexterity in multiple system atrophy. Mov Disord.

[20] Schröter N, Rijntjes M, Urbach H, Weiller C, Treppner M, Kellner E (2022). Disentangling nigral and putaminal contribution to motor impairment and levodopa response in Parkinson’s disease. NPJ Parkinsons Dis.

[21] Rau A, Reisert M, Kellner E, Hosp JA, Urbach H, Demerath T (2021). Increased interstitial fluid in periventricular and deep white matter hyperintensities in patients with suspected idiopathic normal pressure hydrocephalus. Sci Rep.

[22] Rau A, Schroeter N, Blazhenets G, Dressing A, Walter LI, Kellner E (2022). Widespread white matter oedema in subacute COVID-19 patients with neurological symptoms. Brain.

[23] Demerath T, Donkels C, Reisert M, Heers M, Rau A, Schröter N (2021). Gray-white matter blurring of the temporal pole associated with hippocampal sclerosis: a microstructural study involving 3 T MRI and ultrastructural histopathology. Cereb Cortex.

[24] Postuma RB, Berg D, Stern M, Poewe W, Olanow CW, Oertel W (2015). MDS clinical diagnostic criteria for Parkinson’s disease. Mov Disord.

[25] Höglinger GU, Respondek G, Stamelou M, Kurz C, Josephs KA, Lang AE (2017). Clinical diagnosis of progressive supranuclear palsy: the movement disorder society criteria. Mov Disord.

[26] Wenning GK, Stankovic I, Vignatelli L, Fanciulli A, Calandra-Buonaura G, Seppi K (2022). The movement disorder society criteria for the diagnosis of multiple system atrophy. Mov Disord.

[27] Müller J, Wenning GK, Jellinger K, McKee A, Poewe W, Litvan I (2000). Progression of Hoehn and Yahr stages in parkinsonian disorders: A clinicopathologic study. Neurology.

[28] Veraart J, Novikov DS, Christiaens D, Ades-Aron B, Sijbers J, Fieremans E (2016). Denoising of diffusion MRI using random matrix theory. Neuroimage.

[29] Kellner E, Dhital B, Kiselev VG, Reisert M (2016). Gibbs-ringing artifact removal based on local subvoxel-shifts. Magn Reson Med.

[30] Daducci A, Canales-Rodríguez EJ, Zhang H, Dyrby TB, Alexander DC, Thiran J-P (2015). Accelerated microstructure imaging via convex optimization (AMICO) from diffusion MRI data. Neuroimage.

[31] Rolls ET, Huang C-C, Lin C-P, Feng J, Joliot M (2020). Automated anatomical labelling atlas 3. Neuroimage.

[32] Oishi K, Faria A, Jiang H, Li X, Akhter K, Zhang J (2009). Atlas-based whole brain white matter analysis using large deformation diffeomorphic metric mapping: application to normal elderly and Alzheimer’s disease participants. Neuroimage.

[33] Ilinsky I, Horn A, Paul-Gilloteaux P, Gressens P, Verney C, Kultas-Ilinsky K. Human motor thalamus reconstructed in 3D from continuous sagittal sections with identified subcortical afferent territories. 2018. https://www.eneuro.org/content/5/3/ENEURO.0060-18.2018. Accessed 22 Feb 2021. 10.1523/ENEURO.0060-18.2018 PMC6049607 30023427

[34] Vasconcelos N. Feature selection by maximum marginal diversity. Advances in neural information processing systems. 2002. https://papers.nips.cc/paper/2002/hash/bd0cc810b580b35884bd9df37c0e8b0f-Abstract.html. Accessed 11 Nov 2022.

[35] Mitchell T, Archer DB, Chu WT, Coombes SA, Lai S, Wilkes BJ (2019). Neurite orientation dispersion and density imaging (NODDI) and free-water imaging in Parkinsonism. Hum Brain Mapp.

[36] Kamagata K, Hatano T, Okuzumi A, Motoi Y, Abe O, Shimoji K (2016). Neurite orientation dispersion and density imaging in the substantia nigra in idiopathic Parkinson disease. Eur Radiol.

[37] Andica C, Kamagata K, Hatano T, Okuzumi A, Saito A, Nakazawa M (2018). Neurite orientation dispersion and density imaging of the nigrostriatal pathway in Parkinson’s disease: retrograde degeneration observed by tract-profile analysis. Parkinsonism Relat Disord.

[38] Lin C-H, Chen C-M, Lu M-K, Tsai C-H, Chiou J-C, Liao J-R (2013). VBM reveals brain volume differences between parkinson’s disease and essential tremor patients. Front Hum Neurosci.

[39] Mahlknecht P, Hotter A, Hussl A, Esterhammer R, Schocke M, Seppi K (2010). Significance of MRI in diagnosis and differential diagnosis of Parkinson’s disease. Neurodegener Dis.

[40] Talai AS, Sedlacik J, Boelmans K, Forkert ND. Utility of multi-modal MRI for differentiating of parkinson’s disease and progressive supranuclear palsy using machine learning. 2021. https://www.frontiersin.org/articles/10.3389/fneur.2021.648548. Accessed 9 Oct 2022. 10.3389/fneur.2021.648548 PMC8079721 33935946

[41] Shinde S, Prasad S, Saboo Y, Kaushick R, Saini J, Pal PK (2019). Predictive markers for Parkinson’s disease using deep neural nets on neuromelanin sensitive MRI. Neuroimage Clin..

