# Supplementary material for: Complemental Value of Microstructural and Macrostructural MRI in the Discrimination of Neurodegenerative Parkinson Syndromes
Source: Clin Neuroradiol. 2024 Jan 30;34(2):411–20. doi: 10.1007/s00062-023-01377-w (PMC11130007; doi:10.1007/s00062-023-01377-w)
Supplement: Supplementary file 1 — Supplementary Table 1 Results of DeLong’s test within the training cohort Supplementary Table 2 Results of DeLong’s test within the test cohort Supplementary Table 3 Areas under the curve of the receiver operating characteristics in the training cohort Supplementary Table 4 Atlas regions from the AAL 3 atlas for gray matter and the JHU WMPM III atlas for white matter areas with highest variance in the maximum marginal diversity and highest coefficients in the support vector machine with the input combination of Tissue Probability Values + Diffusion Microstructure Imaging [file 62_2023_1377_MOESM1_ESM.docx]

**SUPPLEMENT**

**Supplementary Table 1** Results of DeLong’s test within the training cohort

|  | TPV | DTI | TPV + DTI | NODDI | NODDI  + TPV | NODDI  + DTI | NODDI  + DTI  + TPV | DMI | DMI  + TPV | DMI  + DTI | DMI  + DTI  + TPV | DMI  + NODDI | DMI  + NODDI  + TPV | DMI  + NODDI  + DTI | DMI  + NODDI  + DTI  + TPV |
| --- | --- | --- | --- | --- | --- | --- | --- | --- | --- | --- | --- | --- | --- | --- | --- |
| TPV |  | _X__ | XXX_ | _X__ | XXX_ | _X__ | XX__ | _X__ | _X__ | _X__ | _X__ | _X__ | XX__ | XX__ | XXX_ |
| DTI | _X__ |  | X___ | ____ | X___ | _X_X | X__X | ____ | ___X | ___X | ___X | ____ | ____ | _X_X |  |
| TPV + DTI | XXX_ | X___ |  | ____ | ____ | ____ | ____ | ____ | ____ | ____ | ____ | ____ | ____ |  |  |
| NODDI | _X__ | ____ | ____ |  | ____ | ____ | ____ | ____ | ____ | ____ | ____ | ____ |  |  |  |
| NODDI + TPV | XXX_ | X___ | ____ | ____ |  | ____ | ____ | ____ | ____ | ____ | ____ |  |  |  |  |
| NODDI + DTI | _X__ | _X_X | ____ | ____ | ____ |  | ____ | _X__ | _X__ | ____ |  |  |  |  |  |
| NODDI + DTI + TPV | XXX_ | X__X | ____ | ____ | ____ | ____ |  | __X_ | _X__ |  |  |  |  |  |  |
| DMI | _X__ | ____ | ____ | ____ | ____ | _X__ | __X_ |  |  |  |  |  |  |  |  |
| DMI + TPV | _X__ | ___X | ____ | ____ | ____ | _X__ | _X__ |  |  |  |  |  |  |  |  |
| DMI + DTI | _X__ | ___X | ____ | ____ | ____ | ____ |  |  |  |  |  |  |  |  |  |
| DMI + DTI + TPV | _X__ | ___X | ____ | ____ | ____ |  |  |  |  |  |  |  |  |  |  |
| DMI + NODDI | _X__ | ____ | ____ | ____ |  |  |  |  |  |  |  |  |  |  |  |
| DMI + NODDI + TPV | XX__ | ____ | ____ |  |  |  |  |  |  |  |  |  |  |  |  |
| DMI + NODDI + DTI | _X__ | ___X |  |  |  |  |  |  |  |  |  |  |  |  |  |
| DMI + NODDI + DTI + TPV | XXX_ |  |  |  |  |  |  |  |  |  |  |  |  |  |  |

Each of the four classes (HC/PD/MSA/PSP) was assessed and an "X" indicates a significantly better AUC in the DeLong test whereas „_“ indicates no significant difference.

DMI - Diffusion Microstructure Imaging; DTI - Diffusion Tensor Imaging; NODDI - Neurite Orientation and Dispersion Imaging; TPV - Tissue Probability Value

**Supplementary Table 2** Results of DeLong’s test within the test cohort

|  | TPV | DTI | TPV + DTI | NODDI | NODDI  + TPV | NODDI  + DTI | NODDI  + DTI  + TPV | DMI | DMI  + TPV | DMI  + DTI | DMI  + DTI  + TPV | DMI  + NODDI | DMI  + NODDI  + TPV | DMI  + NODDI  + DTI | DMI  + NODDI  + DTI  + TPV |
| --- | --- | --- | --- | --- | --- | --- | --- | --- | --- | --- | --- | --- | --- | --- | --- |
| TPV |  | ____ | ____ | ____ | _X__ | ____ | ____ | ____ | _X__ | ____ | ____ | ____ | _X__ | ____ | ____ |
| DTI | ____ |  | ____ | ____ | ____ | ____ | ____ | ____ | ____ | ____ | ____ | ____ | ____ | ____ |  |
| TPV + DTI | ____ | ____ |  | ____ | ____ | ____ | ____ | ____ | ____ | ____ | ____ | ____ | ____ |  |  |
| NODDI | ____ | ____ | ____ |  | ____ | ____ | ____ | ____ | ____ | ____ | ____ | ____ |  |  |  |
| NODDI + TPV | _X__ | ____ | ____ | ____ |  | ____ | ____ | ____ | ____ | ____ | ____ |  |  |  |  |
| NODDI + DTI | ____ | ____ | ____ | ____ | ____ |  | ____ | ____ | ____ | ____ |  |  |  |  |  |
| NODDI + DTI + TPV | ____ | ____ | ____ | ____ | ____ | ____ |  | ____ | ____ |  |  |  |  |  |  |
| DMI | ____ | ____ | ____ | ____ | ____ | ____ | ____ |  |  |  |  |  |  |  |  |
| DMI + TPV | _X__ | ____ | ____ | ____ | ____ | ____ | ____ |  |  |  |  |  |  |  |  |
| DMI + DTI | ____ | ____ | ____ | ____ | ____ | ____ |  |  |  |  |  |  |  |  |  |
| DMI + DTI + TPV | ____ | ____ | ____ | ____ | ____ |  |  |  |  |  |  |  |  |  |  |
| DMI + NODDI | ____ | ____ | ____ | ____ |  |  |  |  |  |  |  |  |  |  |  |
| DMI + NODDI + TPV | _X__ | ____ | ____ |  |  |  |  |  |  |  |  |  |  |  |  |
| DMI + NODDI + DTI | ____ | ____ |  |  |  |  |  |  |  |  |  |  |  |  |  |
| DMI + NODDI + DTI + TPV | ____ |  |  |  |  |  |  |  |  |  |  |  |  |  |  |

Each of the four classes (HC/PD/MSA/PSP) was assessed and an "X" indicates a significantly better AUC in the DeLong test whereas „_“ indicates no significant difference.

DMI - Diffusion Microstructure Imaging; DTI - Diffusion Tensor Imaging; NODDI - Neurite Orientation and Dispersion Imaging; TPV - Tissue Probability Value

**Supplementary Table 3** Areas under the curve of the receiver operating characteristics in the training cohort

|  | **Healthy Controls** | **Parkinson’s Disease** | **Multiple System Atrophy** | **Progressive Supranuclear Palsy** |
| --- | --- | --- | --- | --- |
| **TPV** | 0.85 | 0.84 | 0.94 | 0.94 |
| **DTI** | 0.88 | 0.91 | 0.99 | 0.89 |
| **TPV + DTI** | 0.92 | 0.92 | 0.99 | 0.94 |
| **NODDI** | 0.91 | 0.92 | 0.98 | 0.94 |
| **NODDI + TPV** | 0.94 | 0.92 | 0.99 | 0.94 |
| **NODDI + DTI** | 0.92 | 0.95 | 0.98 | 0.96 |
| **NODDI + DTI + TPV** | 0.95 | 0.94 | 0.99 | 0.96 |
| **DMI** | 0.91 | 0.90 | 0.96 | 0.95 |
| **DMI + TPV** | 0.91 | 0.90 | 0.97 | 0.96 |
| **DMI + DTI** | 0.93 | 0.92 | 0.97 | 0.95 |
| **DMI + DTI + TPV** | 0.93 | 0.92 | 0.98 | 0.96 |
| **DMI + NODDI** | 0.92 | 0.93 | 0.97 | 0.96 |
| **DMI + NODDI + TPV** | 0.93 | 0.92 | 0.98 | 0.96 |
| **DMI + NODDI + DTI** | 0.93 | 0.94 | 0.98 | 0.96 |
| **DMI + NODDI + DTI + TPV** | 0.94 | 0.94 | 0.99 | 0.96 |

DMI - Diffusion Microstructure Imaging; DTI - Diffusion Tensor Imaging; NODDI - Neurite Orientation and Dispersion Imaging; TPV - Tissue Probability Value

**Supplementary Table 4** Atlas regions from the AAL 3 atlas for gray matter and the JHU WMPM III atlas for white matter areas with highest variance in the maximum marginal diversity and highest coefficients in the support vector machine with the input combination of Tissue Probability Values + Diffusion Microstructure Imaging

| Maximum marginal diversity |  | Support Vector Machine |  |
| --- | --- | --- | --- |
| Atlas-Region | Variance | Atlas-Region | Coefficient |
| TPV_GM_PUTAMEN_R | 0.36 | DMI_V_extra_GM_Fornix_Stria_terminalis__R | 0.06 |
| TPV_GM_PUTAMEN_L | 0.33 | TPV_WM_Paracentral_Lobule_L | 0.06 |
| TPV_GM_Anterior_limb_of_internal_capsule_R | 0.32 | TPV_WM_Cingulum_Post_R | 0.06 |
| TPV_WM_Inferior_cerebellar_peduncle_R | 0.30 | TPV_GM_Cingulum_Post_R | 0.06 |
| TPV_WM_Medial_lemniscus_R | 0.29 | TPV_WM_Vermis_6 | 0.05 |
| TPV_WM_Middle_cerebellar_peduncle_L | 0.29 | DMI_V_extra_WM_MIDDLE_OCCIPITAL_WM_R | 0.05 |
| TPV_WM_Inferior_cerebellar_peduncle_L | 0.29 | DMI_V_intra_GM_FUSIFORM_WM_R | 0.05 |
| TPV_WM_Lenticular_fasciculus_R | 0.29 | DMI_V_extra_WM_Cerebelum_10_L | 0.05 |
| TPV_WM_Superior_cerebellar_peduncle_R | 0.29 | TPV_GM_Cingulum_Ant_R | 0.05 |
| DMI_V_intra_WM_Middle_cerebellar_peduncle_L | 0.28 | DMI_V_CSF_WM_Pontine_crossing_tract__L | 0.05 |
| DMI_V_extra_WM_Middle_cerebellar_peduncle_R | 0.28 | DMI_V_intra_GM_INFERIOR_TEMPORAL_WM_R | 0.05 |
| TPV_GM_CaudatePutamenVentralStriatum_R | 0.27 | TPV_GM_Inferior_cerebellar_peduncle_R | 0.05 |
| TPV_WM_Cerebral_peduncle_L | 0.27 | TPV_GM_Frontal_Sup_R | 0.05 |
| TPV_GM_Posterior_limb_of_internal_capsule_R | 0.27 | DMI_V_intra_WM_AlFrFxMtPc_R | 0.05 |
| TPV_WM_Pontine_L | 0.27 | DMI_V_extra_WM_Heschl_R | 0.04 |
| TPV_WM_AcBcBsc_L | 0.27 | DMI_V_extra_Cerebellum_9_L | 0.04 |
| TPV_WM_PONS_R | 0.27 | DMI_V_intra_WM_Vermis_10 | 0.04 |
| TPV_WM_Middle_cerebellar_peduncle_R | 0.27 | TPV_GM_Putamen_L | 0.04 |
| TPV_WM_Superior_cerebellar_peduncle_L | 0.27 | DMI_V_CSF_GM_Lingual_R | 0.04 |
| TPV_WM_Lenticular_fasciculus_L | 0.26 | DMI_V_CSF_Angular_R | 0.04 |
| TPV_WM_Midbrain_L | 0.26 | TPV_GM_Superior_fronto_occipial_fasciculus_R | 0.04 |
| TPV_WM_Pontine_crossing_tract_R | 0.26 | TPV_WM_Vermis_10 | 0.04 |
| TPV_WM_SubthalamicNucleus_R | 0.26 | TPV_GM_Medial_geniculate_R | 0.04 |
| TPV_GM_Anterior_limb_of_internal_capsule_L | 0.26 | TPV_GM_Frontal_sup_L | 0.04 |
| TPV_WM_Middle_cerebellar_peduncle_R | 0.25 | DMI_V_extra_WM_MIDBRAIN_L | 0.04 |
| TPV_WM_Pontine_crossing_tract__L | 0.25 | DMI_V_extra_WM_Reticular_Nucl_L | 0.04 |
| TPV_WM_GLOBUS_PALLIDUS_R | 0.25 | TPV_WM_Supp_Motor_Area_R | 0.04 |
| DMI_V_extra_WM_PRECENTRAL__WM_L | 0.25 | DMI_V_CSF_GM_Pallidum_R | 0.04 |
| TPV_WM_Medial_lemniscus_L | 0.25 | DMI_V_intra_WM_Frontal_Inf_Tri_R | 0.04 |
| TPV_WM_Cerebellum_3_R | 0.25 | DMI_V_extra_WM_PRECENTRAL_WM_R | 0.04 |
| TPV_WM_Pallidum_L | 0.24 | DMI_V_CSF_GM_Cingulum_Post_R | 0.04 |
| TPV_WM_Corticospinal_tract_L | 0.24 | TPV_WM_Cerebellum_9_L | 0.04 |
| TPV_WM_Globus_pallidus_medial_R | 0.24 | TPV_WM_Rectus_R | 0.04 |
| TPV_WM_Pontine_R | 0.24 | TPV_GM_Frontal_Inf_Tri_L | 0.04 |
| DMI_V_extra_WM_Supp_Motor_Area_L | 0.24 | TPV_GM_SUPERIOR_FRONTAL_WM_L | 0.04 |
| TPV_WM_MIDBRAIN_L | 0.24 | TPV_WM_Meynerts_Nucl_R | 0.04 |
| TPV_WM_Vermis_1_2 | 0.24 | TPV_WM_Precentral_R | 0.04 |
| TPV_GM_CaudatePutamenVentralStriatum_L | 0.24 | DMI_V_extra_WM_INFERIOR_TEMPORAL__WM_L | 0.04 |
| DMI_V_intra_WM_Cerebellum_8_R | 0.24 | TPV_GM_Temporal_Mid_R | 0.04 |
| TPV_WM_Globus_pallidus_lateral_R | 0.23 | TPV_WM_Cingulum_Mid_L | 0.04 |
| TPV_WM_Basal_forebrain_R | 0.23 | DMI_V_extra_WM_LINGUAL_L | 0.04 |
| TPV_WM_SubthalamicNucleus_L | 0.23 | DMI_V_CSF_WM_lateral_Geniculate_R | 0.03 |
| DMI_V_extra_WM_Cerebellum_4_5_R | 0.23 | DMI_V_intra_WM_Cerebellum_3_L | 0.03 |
| DMI_V_intra_WM_Middle_cerebellar_peduncle_R | 0.23 | DMI_V_intra_Inferior_fronto_occipital_fasciculus_R | 0.03 |
| TPV_WM_CentromedianParafascicular_Nucl_L | 0.23 | TPV_GM_Frontal_Inf_Orb_L | 0.03 |
| DMI_V_intra_WM_Cerebellum_4_5_R | 0.23 | DMI_V_extra_WM_Reticular_Nucl_R | 0.03 |
| DMI_V_extra_WM_Cerebellum_8_R | 0.23 | DMI_V_intra_WM_Cerebellum_10_R | 0.03 |
| TPV_WM_GLOBUS_PALLIDUS_L | 0.23 | TPV_WM_Precentral_L | 0.03 |
| TPV_WM_Corticospinal_tract_L | 0.23 | TPV_WM_Frontal_Inf_Oper_L | 0.03 |
| DMI_V_extra_WM_Cerebellum_8_L | 0.23 | DMI_V_intra_GM_Ventral_anterior_pallidal_L | 0.03 |

| Maximum marginal diversity |  | Support Vector Machine |  |
| --- | --- | --- | --- |
| Atlas-Region | Variance | Atlas-Region | Coefficient |
| TPV_GM_PUTAMEN_R | 0.36 | TPV_WM_Vermis_6 | 0.061 |
| TPV_GM_PUTAMEN_L | 0.33 | NODDI_OD_WM_rh_VPi | 0.056 |
| TPV_GM_Anterior_limb_of_internal_capsule_R | 0.32 | DTI_FA_GM_Vermis_4_5 | 0.049 |
| TPV_WM_Inferior_cerebellar_peduncle_R | 0.30 | TPV_GM_Inferior_cerebellar_peduncle_right | 0.048 |
| TPV_WM_Medial_lemniscus_R | 0.29 | DTI_mD_GM_FUSIFORM_WM_right | 0.048 |
| NODDI_ICVF_WM_Cerebrellum_4_5_L | 0.29 | DTI_FA_WM_MIDDLE_OCCIPITAL_WM_right | 0.046 |
| TPV_WM_Middle_cerebellar_peduncle_L | 0.29 | DTI_rD_WM_Corticospinal_tract_right | 0.042 |
| TPV_WM_Inferior_cerebellar_peduncle_L | 0.29 | DTI_FA_WM_Fornix_Stria_terminalis_left | 0.040 |
| TPV_WM_Lenticular_fasciculus_R | 0.29 | DTI_rD_WM_Corticospinal_tract_left | 0.039 |
| NODDI_ICVF_WM_Cerebellum_4_5_R | 0.29 | DTI_FA_WM_Vermis_10 | 0.038 |
| TPV_WM_Superior_cerebellar_peduncle_R | 0.28 | DTI_FA_GM_Occipital_Mid_R | 0.038 |
| DTI_mD_WM_Cerebellum_8_L | 0.28 | DTI_FA_WM_NucleusLimitans_R | 0.037 |
| NODDI_ICVF_WM_Cerebelum_8_R | 0.28 | NODDI_ICVF_GM_VentralAnteriorPallidalNucleus_L | 0.037 |
| NODDI_ICVF_WM_Middle_cerebellar_peduncle_L | 0.28 | NODDI_OD_WM_ NucleusLimitans_R | 0.036 |
| TPV_GM_CaudatePutamenVentralStriatum_R | 0.27 | DTI_FA_GM_INFERIOR_TEMPORAL_WM_right | 0.036 |
| TPV_WM_Cerebral_peduncle_L | 0.27 | TPV_WM_Paracentral_Lobule_L | 0.036 |
| TPV_GM_Posterior_limb_of_internal_capsule_R | 0.27 | TPV_WM_Precentral_R | 0.036 |
| NODDI_ICVF_WM_Vermis_4_5 | 0.27 | DTI_rD_GM_inferior_fronto_occipital_fasciculus_right | 0.036 |
| TPV_WM_Pontine_L | 0.27 | DTI_FA_WM_PRECENTRAL_WM_right | 0.035 |
| TPV_WM_AcBcBsc_L | 0.27 | NODDI_OD_WM_STN_L | 0.034 |
| TPV_WM_PONS_R | 0.27 | NODDI_OD_GM_Insula_L | 0.034 |
| NODDI_OD_GM_PUTAMEN_R | 0.27 | NODDI_OD_GM_Frontal_Sup_Medial_R | 0.033 |
| NODDI_ICVF_WM_Cerebellum_6_R | 0.27 | DTI_aD_GM_VentralPosteriorLateralMedialNucleus_R | 0.033 |
| TPV_WM_Superior_cerebellar_peduncle_L | 0.26 | TPV_WM_Rectus_L | 0.033 |
| TPV_WM_Lenticular_fasciculus_L | 0.26 | DTI_aD_WM_PRECUNEUS_WM_left | 0.033 |
| NODDI_ICVF_WM_Cerebellum_8_L | 0.26 | TPC_GM_Frontal_Sup_L | 0.033 |
| TPV_WM_Midbrain_L | 0.26 | NODDI_OD_WM_RedNucleus_L | 0.033 |
| TPV_WM_Pontine_crossing_tract_R | 0.26 | DTI_FA_WM_Cerebelum_3_L | 0.033 |
| TPV_WM_SubthalamicNucleus_R | 0.26 | NODDI_ICVF_WM_MIDBRAIN_left | 0.032 |
| TPV_GM_Anterior_limb_of_internal_capsule_L | 0.26 | DTI_FA_WM_Middle_cerebellar_peduncle_right | 0.032 |
| NODDI_ICVF_WM_Middle_cerebellar_peduncle_right | 0.26 | TPV_WM_Precentral_L | 0.032 |
| TPV_WM_Middle_cerebellar_peduncle_R | 0.25 | TPV_GM_ AcBcBsc_L | 0.032 |
| TPV_WM_Pontine_crossing_tract__L | 0.25 | DTI_mD_WM_Corticospinal_tract_right | 0.031 |
| NODDI_ICVF_WM_Cerebellum_Crus1_R | 0.25 | NODDI_ISOVF_GM_Vermis_3 | 0.031 |
| TPV_WM_GLOBUS_PALLIDUS_R | 0.25 | TPV_WM_Cingulum_Post_R | 0.031 |
| DTI_mD_WM_Middle_cerebellar_peduncle_left | 0.25 | NODDI_OD_GM_CaudatePutamenVentralStriatum_R | 0.031 |
| DTI_FA_WM_Cerebellum_9_R | 0.25 | TPV_GM_Frontal_Inf_Tri_L | 0.030 |
| TPV_WM_Medial_lemniscus_L | 0.25 | TPV_GM_Cingulum_Post_R | 0.030 |
| TPV_WM_Cerebelum_3_R | 0.25 | DTI_aD_WM_lh_alfrfxmtpc | 0.030 |
| NODDI_ICVF_WM_Superior_corona_radiata_left | 0.24 | TPV_WM_Cingulum_Mid_L | 0.030 |
| TPV_WM_Pallidum_L | 0.24 | NODDI_OD_GM_AnteriorNuclei_L | 0.030 |
| DTI_rD_GM_Cerebellum_4_5_R | 0.24 | DTI_rD_GM_Connectivetissue_R | 0.029 |
| TPV_WM_Corticospinal_tract_L | 0.24 | DTI_FA_WM_Cerebelum_10_L | 0.029 |
| TPV_WM_Globus_pallidus_medial_R | 0.24 | TPV_GM_PUTAMEN_left | 0.029 |
| TPV_WM_Pontine_R | 0.24 | DTI_rD_WM_Pontine_L | 0.029 |
| TPV_WM_MIDBRAIN_L | 0.24 | DTI_rD_GM_INFERIOR_TEMPORAL_WM_right | 0.029 |
| DTI_aD_WM_Middle_cerebellar_peduncle_left | 0.24 | TPV_WM_MIDDLE_FRONTOORBITAL_WM_right | 0.029 |
| TPV_WM_Vermis_1_2 | 0.24 | TPV_GM_Putamen_L | 0.029 |
| NODDI_ICVF_WM_Frontal_Inf_Oper_L | 0.24 | NODDI_OD_GM_PUTAMEN_right | 0.029 |
| NODDI_ICVF_WM_Cerebelum_6_L | 0.24 | DTI_mD_WM_Corticospinal_tract_left | 0.029 |

**Supplementary Table 5** Atlas regions from the AAL 3 atlas for gray matter and the JHU WMPM III atlas for white matter areas with highest variance in the maximum marginal diversity and highest coefficients in the support vector machine with the input combination of Tissue Probability Values + Diffusion Tensor Imaging + Neurite Orientation Dispersion and Density Imaging
